# Laparoscopic Repair of Duodenal Perforation Using the Falciform Ligament: A Cross-Sectional Study

**DOI:** 10.7759/cureus.73576

**Published:** 2024-11-13

**Authors:** Ganugapanta Prem Sai Reddy, Alexander Mecheri Antony, Ramalakshmi Venkateswaran

**Affiliations:** 1 General Surgery, Sree Balaji Medical College and Hospital, Chennai, IND

**Keywords:** duodenal perforation, falciform ligament, laparoscopic repair, minimally invasive surgery, surgical outcomes

## Abstract

Background

Duodenal perforation is a life-threatening condition. Laparoscopic repair using the falciform ligament is a minimally invasive technique that has shown promising results. We present a case series of patients who underwent laparoscopic repair of duodenal perforation using the falciform ligament as an alternative to conventional techniques.

Methodology

The present study was a cross-sectional study carried out in the Department of General Surgery, Sree Balaji Medical College and Hospital, Chennai, India, between January 2023 and August 2024. The study was carried out among all the patients who underwent laparoscopic repair of duodenal perforation using the falciform ligament. The data collection was done by the principal investigator, himself, using a semi-structured proforma.

Results

The mean operative time among the participants was 86.25 ± 17.07 minutes. The mean time to start liquids was 21.42 ± 3.26 hours, and the mean time to start solids was 37.67 ± 2.06 hours. No post-operative complications were reported or identified. The post-operative pain score was 2.55 ± 1.31. The mean post-operative satisfaction score was 8.59 ± 2.84. The mean duration of hospital stay was 5.51 ± 1.21 days. No participants had any complications or died during the follow-up period. The technical success rate was 100%.

Conclusion

Laparoscopic repair of duodenal perforation using the falciform ligament is a feasible and effective technique. This approach offers minimal invasiveness, reduced morbidity, and faster recovery. Our case series demonstrates the efficacy of this technique in managing duodenal perforation.

## Introduction

The perforation of the duodenum is a potentially life-threatening condition [1]. The perforations can be spontaneous or traumatic. The spontaneous ones are mainly due to peptic ulcer disease [2]. *Helicobacter pylori* infections and non-steroidal anti-inflammatory agents' overuse have been the common underlying causes of peptic ulcer disease, leading to perforations [3]. About 5% of peptic ulcers end up with perforation [4]. Vergara et al. reported physiological stress, corticosteroids, smoking, and a previous history of ulcer disease as the risk factors leading to perforation of a peptic ulcer [5]. Iatrogenic perforations occur either during an endoscopic procedure, or they can be an operative injury [6,7].

Laboratory tests, like serum amylase level, gastrin level, and C-reactive protein, can be done to rule out another diagnosis [8]. Arterial blood gas analysis can be done to evaluate the metabolic compromise in case the patient is septic [9]. One can go for an upright chest radiograph, which shows free air below the diaphragm in case of perforation. The radiographic test that has higher diagnostic accuracy (98%) is a non-contrast computed tomography (CT) scan [10]. For the diagnosis of duodenal perforation, double-contrast CT is the most useful technique [11].

The management of perforation will be determined by the perforation type. Both contained and non-contained perforations are possible. Whereas the contents of the latter leak into the abdominal cavity, the former's neighboring organs stop the free leakage. Conservative treatment is an option for a contained perforation, but, in the latter case, surgery is the best course of action [1]. For minor non-contained perforations, the mode of management is either endoscopic or simple surgical; for major non-contained perforations, duodenoduodenostomy, duodenojejunostomy, and Bilroth II operation can be performed [12]. In general, either an open or laparoscopic omental patch closure is the most often performed procedure. The viability and availability of the same determine if using the larger omentum is feasible. The falciform ligament was suggested as a substitute in those situations [13]. The objective of the current research was to evaluate the technical success rate of laparoscopic repair of duodenal perforation using the falciform ligament, as well as the safety and effectiveness of this technique in managing duodenal perforation. Post-operative outcomes, such as mortality and hospital stay, were also evaluated.

## Materials and methods

The present study was a cross-sectional study carried out in the Department of General Surgery, Sree Balaji Medical College and Hospital, Chennai, India, between January 2023 and August 2024. The study was carried out among all the patients who underwent laparoscopic repair of duodenal perforation using the falciform ligament, while those who underwent either open or other surgical procedures were excluded. Patients between the ages of 18 and 60 years, from both sexes, were included as participants. The ethical clearance for the study was obtained from the institutional ethics committee, and informed consent was obtained from all the participants included in the study. Data collection was done by the principal investigator himself using a semi-structured proforma.

**Figure 1:**
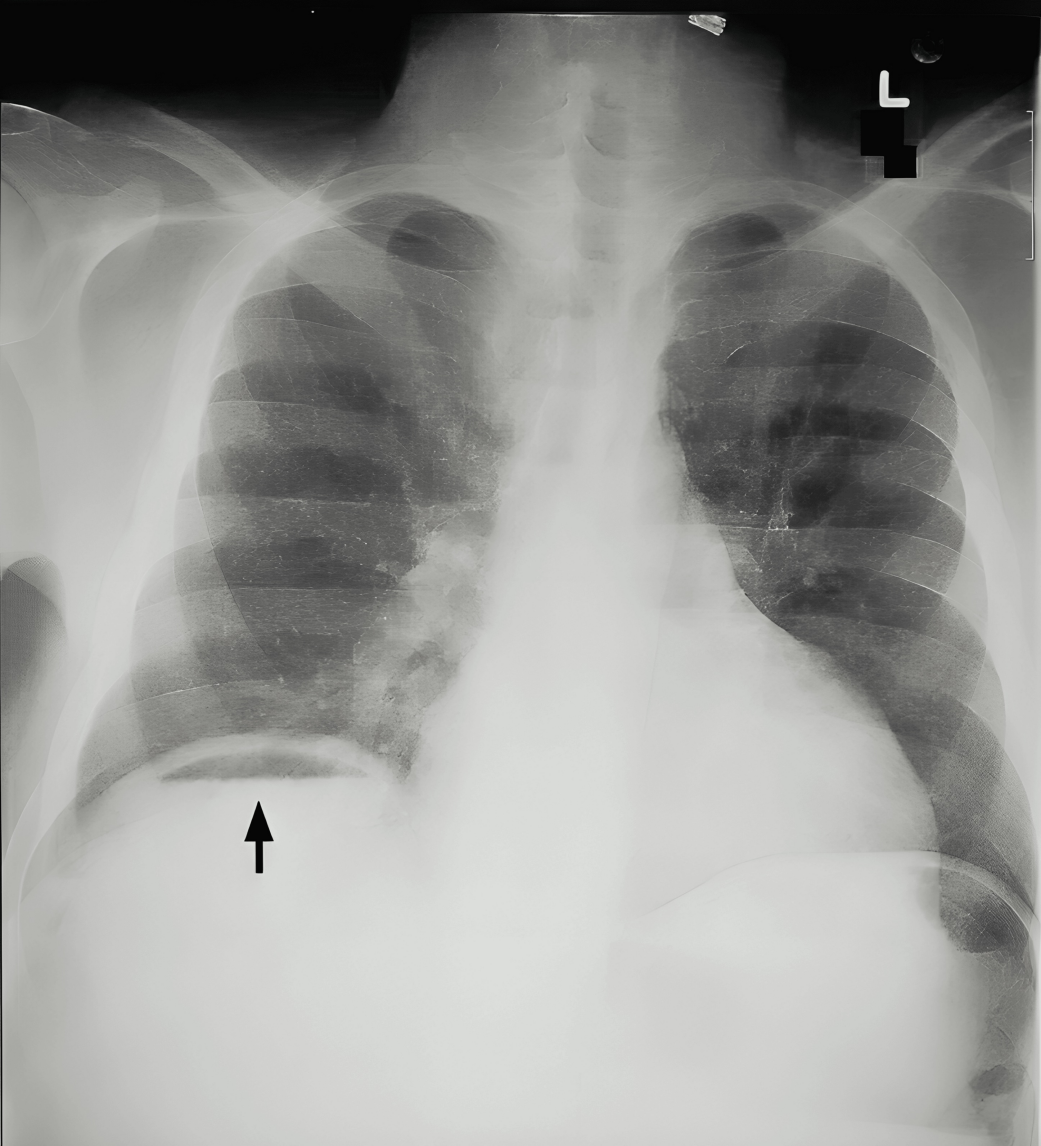
Air below diaphragm (marked with black arrow)

**Figure 2:**
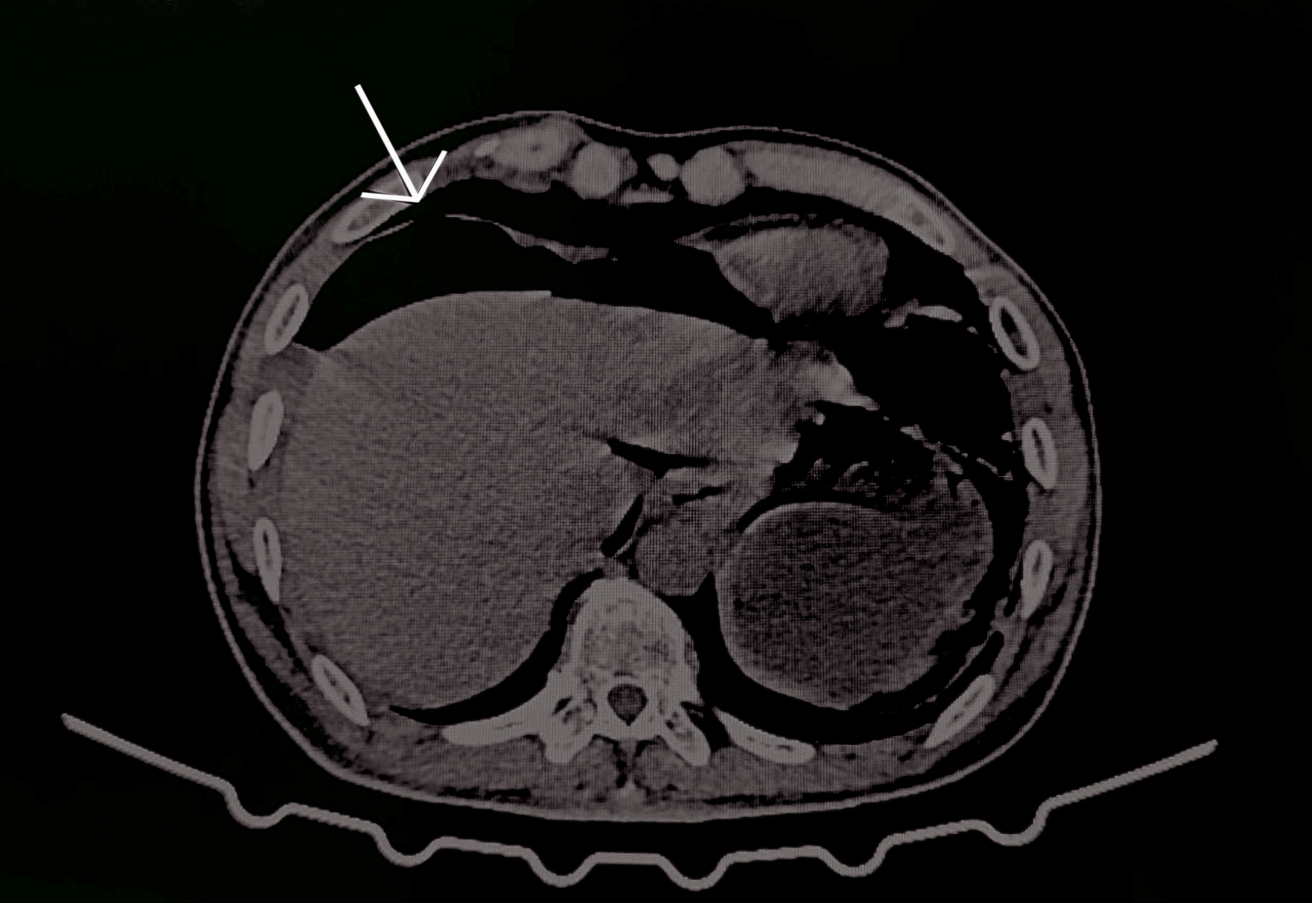
CT image showing D1 perforation (marked with white arrow) CT, computed tomography; D1, duodenum 1

The age and sex of the participants were recorded initially, after obtaining informed consent. The co-morbidities, if any, present were sought for, and the specifics were recorded. The presenting complaint of the participants was obtained from the case sheets, along with the clinical findings, such as distension of the abdomen, tenderness, rigidity, and nature of bowel sounds (absent) at the time of admission. If the participants had undergone radiological tests, such as X-ray (Figure 1) and CT (Figure 2), their findings specific to the perforation, if any, were noted down. The total leukocyte count and the hemoglobin estimates, as obtained from the hematological laboratory, were recorded. The American Society of Anesthesiologists (ASA) category of the patient was also noted.

**Figure 3:**
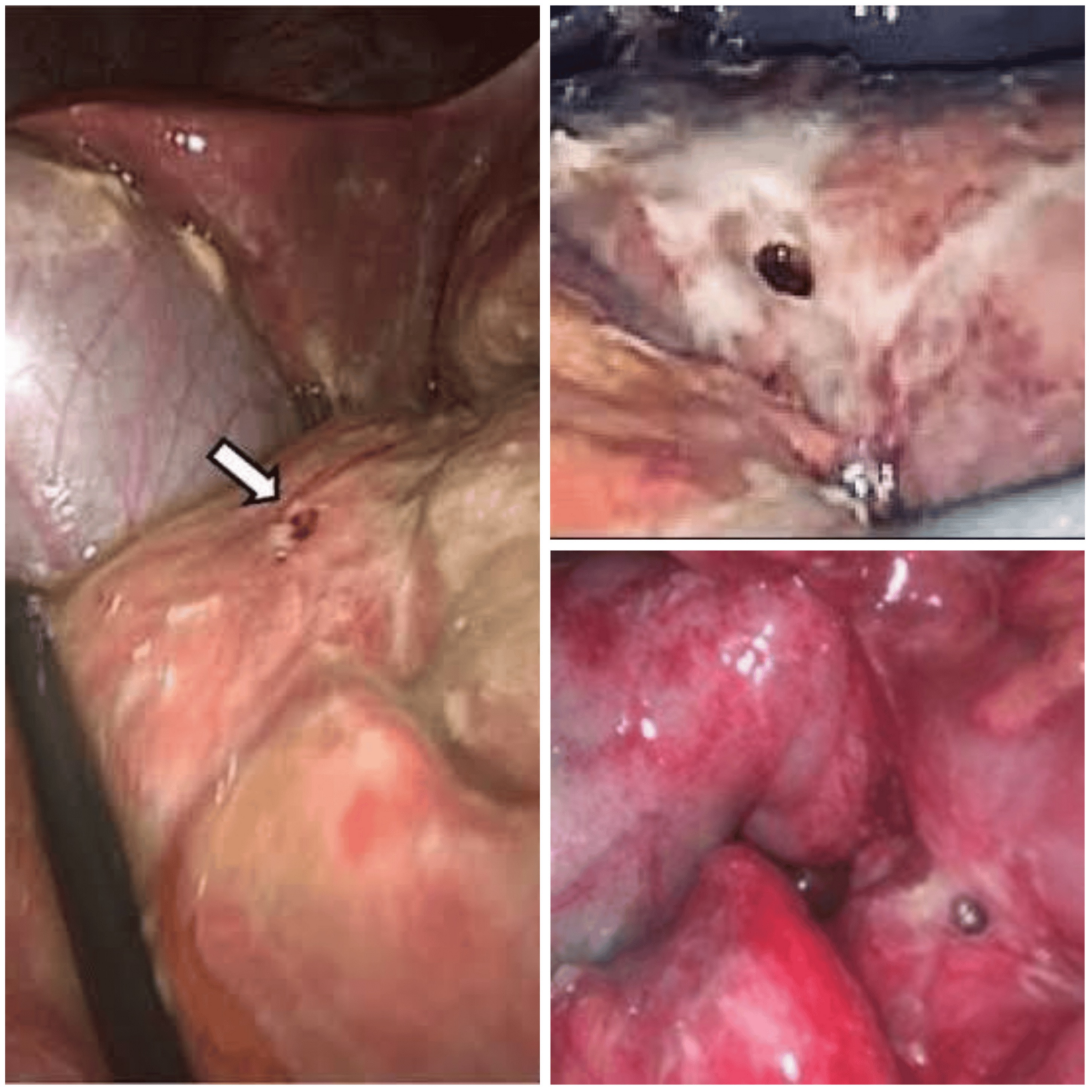
Ulcer appeared on the anterior aspect of the first part of duodenum (white arrow)

**Figure 4:**
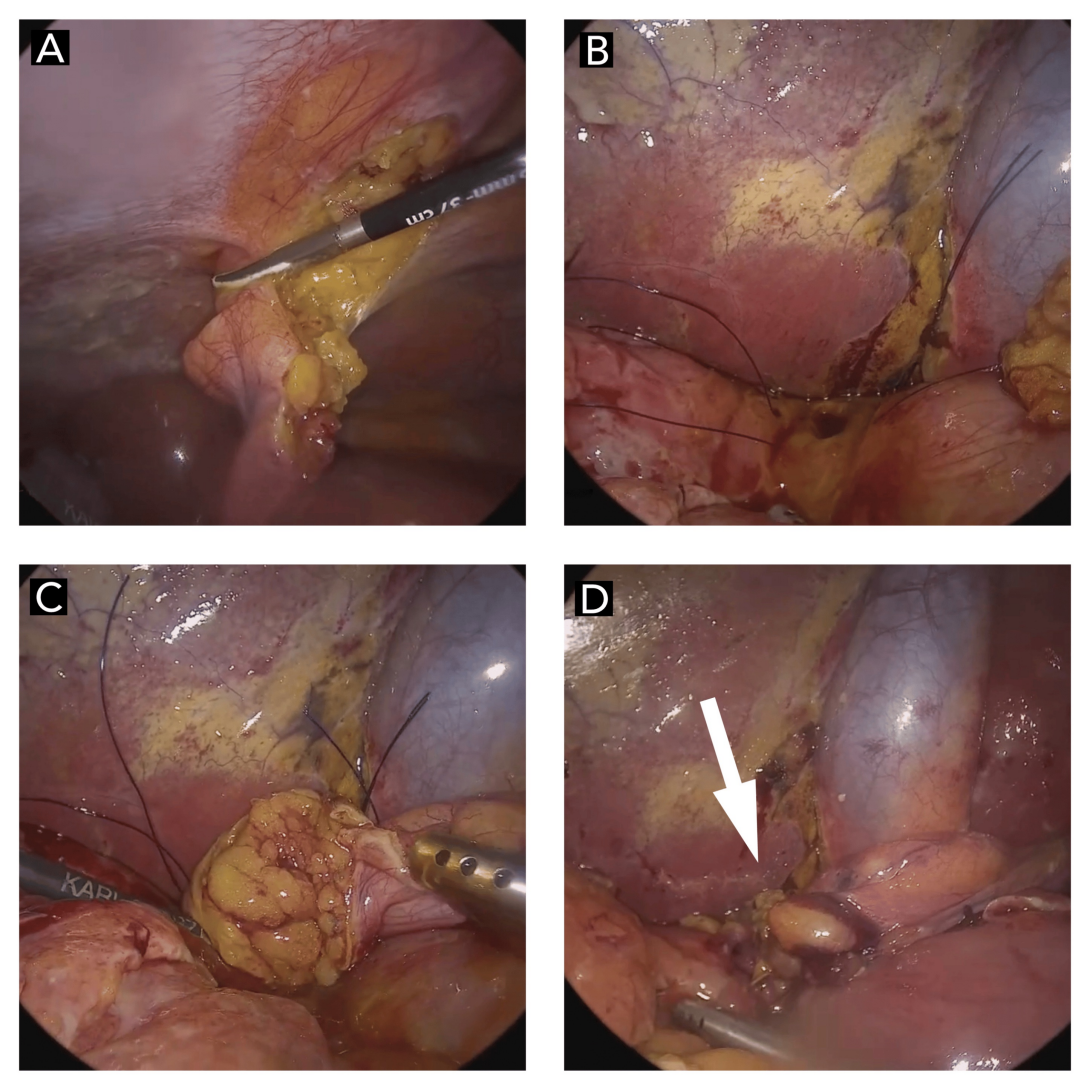
Intraoperative findings (A) Mobilization of the falciform ligament; (B) Intraoperative view showing perforation of the first part of the duodenum; (C-D) Falciform ligament patch over perforated duodenal ulcer (white arrow)

The following were the surgical steps while performing a laparoscopic repair of duodenal perforation using the falciform ligament: under general anesthesia with a nasogastric tube, the patient is placed in a supine position on the operating table, with the legs in stirrups, the knees slightly bent, and the hips flexed approximately 10°. The operating table is tilted head-up by approximately 15°. A compression bandage is used on the legs during the operation to prevent thromboembolism. The surgeon stands between the patient's legs. The first assistant, whose main task is to position the video camera, stands on the patient’s left side. Three ports are used: a 10 mm umbilical port, a 10 mm left port, and a 5 mm right upper quadrant trocar. Initially, the peritoneal cavity is explored, lavage is done, and peritoneal fluid is sent for culture. Exploration of the abdominal cavity is then performed in order to visualize the perforation. After identifying the duodenal perforation (Figure 3), metallic suction is introduced, and the edges are freshened (the standard tip size of the Maryland dissector is 10 mm circumferentially, which is used to measure the radius of the perforation). The duodenal ulcer is sutured with two absorbable surgical sutures. The ends of the sutures are left long inside the abdominal cavity, with the needles carefully positioned on the greater omentum to avoid organ injury. Then, the falciform ligament is released to allow tension-free mobilization and fixed to the duodenal perforation using 3-0 prolene (Figure 4). At the end of the surgical procedure, a suction drain is placed through the 5 mm port in the right iliac fossa, which is positioned close to the flap.

The operative time of the participants, as recorded in the case sheets, was noted down, followed by the time to start liquids and solids, respectively. The operative time was recorded in minutes, while the time to start liquids or solids was recorded in hours. The post-operative complications, if any, were noted. The post-operative pain score and satisfaction score were obtained using a visual analog scale. The duration of hospital stay was noted down.

Any findings among the participants during the follow-up period were also recorded. The participants, once discharged, were followed up in the second week, third month, and sixth month, and asked for any signs and symptoms related to complications.

The data collected were entered into Microsoft Excel 2019 (Microsoft® Corp., Redmond, WA, USA), and the master chart was created. The master chart was then loaded onto Epi Info version 7.2 (Centers for Disease Control and Prevention (CDC), Atlanta, GA, USA). Both quantitative and categorical variables were present in the study. To summarize the quantitative variables, the mean and standard deviation were used. When it was required to know the distribution of frequency among the various categories of quantitative variables, they were converted to categorical variables and summarized using frequency and percentages.

## Results

There were 12 patients included, with the mean age among the participants in the present study being 40.58 ± 6.44 years. Five (41.7%) were males. Three (25%) had pre-existing acid peptic disease, three (25%) had hyperlipidemia with metabolic dysfunction associated with steatotic liver disease, and three (25%) had diabetes (Table 1).

**Table 1:** Distribution according to age, sex and presence of comorbidities N%, frequency; MASLD, metabolic dysfunction associated steatotic liver disease; DM, diabetes mellitus; OHA, oral hypoglycaemic agents

Variable		N (%)
Mean age (in years)		40.58 ± 6.44
Sex	Male	5 (41.7)
	Female	7 (58.3)
Comorbidities	Acid peptic disease	3 (25)
	Hyperlipidemia MASLD	3 (25)
	DM on OHA	3 (25)
	Nil	3 (25)

With regard to the presenting complaints, six (50%) complained of epigastric pain alone, while six (50%) complained of both epigastric pain and constipation. On examination, none had bowel sounds, nine (75%) had a distended abdomen, the abdomen was tender in nine (75%), and in three (25%), the abdomen was rigid. On X-ray, six (50%) had air under the diaphragm, and in CT, six (50%) had D1 perforation. The mean total cell count was 18774.83 ± 2245.85 cells per cu.mm, and the mean hemoglobin level was 11.65 ± 0.95 gm% (Table 2).

**Table 2:** Distribution according to presenting symptoms, clinical findings per abdomen, radiological findings and hematological parameters N%, frequency; CT, computed tomography; D1, duodenum 1

Variable		N (%)
Presenting complaints	Epigastric pain and constipation	6 (50)
	Epigastric pain	6 (50)
Clinical examination findings	Distended abdomen	9 (75)
	Tender	9 (75)
	Rigidity	3 (25)
	Bowel sounds absent	12 (100)
X-ray	Air under diaphragm	6 (50)
CT	D1 perforation	6 (50)
Hematological parameters	Total count (cells per cu.mm)	18774.83 ± 2245.85
	Hemoglobin (gm%)	11.65 ± 0.95

With regard to ASA, five (41.7%) had an ASA score of 2, and seven (58.3%) had an ASA score of 3. Three (25%) had a perforation of 1 cm, a perforation of size 1.5 cm was found in three (25%), and perforations of size 1.75 cm and 2 cm were found in two (16.7%), respectively. Two (16.7%) had adhesions as added difficulties (Table 3).

**Table 3:** Distribution according ASA categories and intraoperative findings among the participants N%, frequency; ASA, American Society of Anesthesiologists

Variable		N (%)
ASA	2	5 (41.7)
	3	7 (58.3)
Size of the perforation (in cms)	1	3 (25)
	1.5	3 (25)
	1.75	3 (25)
	2	3 (25)
Added difficulties	Adhesions	2 (16.7)
	Nil	10 (83.3)

About six participants had a total operative time between 60 and 89 minutes, and another six participants had an operative time between 90 and 119 minutes. The mean operative time among the participants was 86.25 ± 17.07 minutes. None of the participants required conversion to open surgery. For six (50%) participants, liquids were started at 20 to 22 hours, while for three (25%) participants, it was started at 16 to 19 hours, and for another three (25%) participants, it was started at 23 to 26 hours, respectively. The mean time to start liquids was 21.42 ± 3.26 hours, and the mean time to start diet was 37.67 ± 2.06 hours. For seven (58.3%) participants, diet was started at 38 to 40 hours, and for five (41.6%) participants, it was started at 34 to 37 hours. No post-operative complications were reported or identified among the participants. The post-operative pain score, as reported by the participants, was 2.55 ± 1.31. The mean post-operative satisfaction score was 8.59 ± 2.84. Nine (75%) participants stayed in the hospital for four to five days, and three (25%) stayed in the hospital for six to seven days. The mean duration of hospital stay was 5.51 ± 1.21 days (Table 4).

**Table 4:** Distribution according to operating time, time to start liquids, time to start solids and postoperative complications among the participants N%, frequency

Variable			N (%)
Total operative time (in minutes)		60-89	6 (50)
		90-119	6 (50)
Time of starting liquids (in hours)		16-19	3 (25)
		20-22	6 (50)
		23-26	3 (25)
Time of starting diet (in hours)		34-37	5 (41.6)
		38-40	7 (58.3)
Post-operative complications		Nil	12 (100)
Post-operative pain score			2.55 ± 1.31
Post-operative satisfaction score			8.59 ± 2.84
Duration of hospital stay (in days)	4-5		9 (75)
	6-7		3 (25)

## Discussion

The perforation of the duodenum is a potentially life-threatening condition that poses a high risk of mortality among surgical emergency conditions. The management of perforation will be determined by the type of perforation. A perforation can be either contained or non-contained [1]. For minor non-contained perforations, the mode of management is either endoscopic or simple surgical [12]. In general, the most commonly performed procedure is an omental patch closure, but the feasibility of the procedure varies with the availability and viability of the greater omentum. In those circumstances, it has been proposed to use the falciform ligament as an alternative [12]. The technique was initially put forth by Fry et al. [14]. Li et al., in a paper about the morphology of the falciform ligament, stated that both the left phrenic artery and the middle segment artery supply blood to the falciform ligament, making it a well-vascularized structure [15]. Munro et al., in 1996, were the first to effectively use the falciform ligament patch for the laparoscopic repair of duodenal perforation [16].

The present cross-sectional study was carried out to assess the technical success rate of laparoscopic repair of duodenal perforation using the falciform ligament, to evaluate the post-operative outcomes, including mortality and hospital stay, and to assess the safety and efficacy of this technique in managing duodenal perforation. This study included 12 patients who were admitted to the Department of General Surgery with duodenal perforation and underwent laparoscopic repair using the falciform ligament between January 2023 and August 2024.

The strengths of this study are as follows: Novel Approach: the use of the falciform ligament as a patch for laparoscopic repair of duodenal perforation is a novel approach that has not been extensively studied; High Technical Success Rate: the study reported a high technical success rate of 100%, indicating that the procedure was successful in all patients; Low Morbidity: the study reported low morbidity, with no major complications or conversions to open surgery; Short Hospital Stay: the mean hospital stay was 5.51 days, which is relatively short compared to other studies; Low Pain Scores: the post-operative pain scores were low, indicating that the patients had minimal pain; High Satisfaction Scores: the post-operative satisfaction scores were high, indicating that the patients were satisfied with the outcome; No Recurrences: there were no recurrences of the perforation during the follow-up period; Limited Generalizability: the study only included patients with duodenal perforation, which may limit the generalizability of the results to other types of perforations.

The follow-up duration for laparoscopic repair of duodenal perforation using the falciform ligament is typically as follows: immediate post-operative period: 24-48 hours, early post-operative period: 1-2 weeks, late post-operative period: 6-12 weeks, long-term follow-up: 6-12 months.

During these follow-up periods, the patient is monitored for: signs of complications (e.g., infection, bleeding, leakage), abdominal pain or discomfort, digestive issues (e.g., nausea, vomiting, diarrhea), nutritional status, and quality of life.

The mean operative time among the participants was 86.25 ± 17.07 minutes. None of the participants required conversion to open surgery. For six (50%) participants, liquids were started at 20 to 22 hours, while for three (25%) participants, it was started at 16 to 19 hours and 23 to 26 hours, respectively. The mean time to start liquids was 21.42 ± 3.26 hours. For seven (58.3%) participants, diet was started at 38 to 40 hours, and for five (41.6%) participants, it was started at 34 to 37 hours. The mean time to start diet was 37.67 ± 2.06 hours. No post-operative complications were reported or identified among the participants. The post-operative pain score, as reported by the participants, was 2.55 ± 1.31. The mean post-operative satisfaction score was 8.59 ± 2.84. The above represents that the patient had minimal pain. Nine (75%) participants stayed in the hospital for four to five days, and three (25%) participants stayed in the hospital for six to seven days. The mean duration of hospital stay was 5.51 ± 1.21 days.

No participants had any complications or died during the follow-up period. All participants had follow-up visits in the second week, third month, and sixth month. No reoperation or readmission was required for the participants. The technical success rate was 100%, with a 95% CI of 85% to 100%. No participant had any recurrence during the follow-up period. Elgazar et al. also reported a similar result of no complications during the intraoperative and follow-up periods among the recipients of laparoscopic repair using the falciform ligament [13]. Boshnaq et al. reported good postoperative recovery with the help of the falciform ligament patch [17]. The use of the falciform ligament as a patch offers several advantages, including the minimally invasive approach, reduced morbidity, faster recovery, and the avoidance of mesh placement.

The strength of the article is that it adds valuable information about the utilization of falciform ligament patches in laparoscopic repair as an alternative technique to the existing ones.

## Conclusions

Duodenal perforation can be safely and effectively managed with the use of the falciform ligament as a patch, which has a quick recovery period and a minimal risk of complications. This alternative strategy is practical and efficient, and it has a number of benefits over conventional approaches. Falciform ligament repair demonstrated a high technical success rate, good post-operative results, and a minimally invasive technique, as demonstrated by this case series.
